# Ethics of genomic passports: should the genetically resistant be exempted from lockdowns and quarantines?

**DOI:** 10.1136/medethics-2021-107297

**Published:** 2021-06-25

**Authors:** Christopher Gyngell, Julian Savulescu

**Affiliations:** 1 Biomedical Ethics Research Group, Murdoch Childrens Research Institute, Parkville, Victoria, Australia; 2 Department of Paediatrics, The University of Melbourne, Melbourne, Victoria, Australia; 3 Faculty of Philosophy, University of Oxford, Oxford, UK

**Keywords:** public health ethics, COVID-19, genetic information, history of health ethics/bioethics

## Abstract

Lockdowns and quarantines have been implemented widely in response to the COVID-19 pandemic. This has been accompanied by a rise in interest in the ethics of ‘passport’ systems that allow low-risk individuals greater freedoms during lockdowns and exemptions to quarantines. Immunity and vaccination passports have been suggested to facilitate the greater movement of those with acquired immunity and who have been vaccinated. Another group of individuals who pose a low risk to others during pandemics are those with genetically mediated resistances to pathogens. In this paper, we introduce the concept of genomic passports, which so far have not been explored in the bioethics literature. Using COVID-19 as an illustrative example, we explore the ethical issues raised by genomic passports and highlight differences and similarities to immunity passports. We conclude that, although there remain significant practical and ethical challenges to the implementation of genomic passports, there will be ways to ethically use them in the future.

## Introduction

### COVID-19 and immunity passports

The COVID-19 pandemic has seen the impositions of restrictive public health measures around the world, including forced lockdowns, quarantines and limitations on the use of public spaces. In the absence of a vaccine, restricting the movement of individuals is one of the most effective ways to slow the spread of a virus. However, restrictions on movement exact a high toll on individuals and have negative impacts on society. Being confined at your home, or in a hotel room, is a contributory factor to mental illness, including anxiety and depression.^1^ Furthermore, lockdowns often prevent or discourage people from accessing treatment for unrelated conditions, leading to increased deaths from cancer^2^ and other disease.^3^ More broadly, reduced movement correlates with reduced economic activity and therefore impacts many aspects of society.^4^

Significantly, the cost associated with lockdowns disproportionately harms members of a community who are already the worst-off. This includes those with insecure employment,^5^ heavy caring responsibilities^6^ and without financial and personal safety nets.^7^

Lockdowns and quarantines are often justified on the basis that they are the least restrictive way possible to prevent outbreaks.^8^ Because we cannot know who in a community is carrying the virus, allowing the free movement of any individual increases the net risk to all. Some models indicate that lockdowns implemented in Europe in response to COVID-19 averted over 3 million deaths.^9^ Furthermore, lockdowns can lead to virus eradication and expedite a return to everyday life for a society and its citizens.^10^ Policies that restrict the freedom of movement of all members of a society can be seen as justified, given their potential to avert great harm.

However, suppose it becomes possible to identify individuals who genuinely pose no (or minimal) risk to others? Restricting the movement of these individuals is difficult to justify. As there is no risk of these individuals spreading the virus to others, subjecting such individuals to a lockdown or quarantine imposes a cost on them for no benefit. If these individuals are significantly harmed as a consequence of lockdown, it can be seen as a significant injustice.

One group of people who likely pose no, or very low, risk to others during a pandemic are those who have acquired immunity.^11^ This has led to interest in so-called ‘immunity passports’ or ‘immunity certificates’. Immunity passports could take different forms such as wristbands, smartphone apps or physical certificates and provide a mechanism for individuals with an acquired resistance to be granted greater freedom of movement. During periods of ‘lockdown’, immunity passports could allow immune individuals to follow less stringent requirements around restrictions of movement and social distancing. The governments of UK, Estonia, Indonesia and Chile have considered implementing immunity passports, assuming a reliable test for COVID-19 antibodies is developed.^12^ Vaccination passports would offer similar freedoms to those who have received COVID-19 vaccines.

### Genomic passports

Another group of people who pose low or no risk to others during a pandemic are those that are genetically resistant to the virus. For many infectious diseases, some portion of the population have a genetically mediated resistance.^13^ This is also true of COVID-19. Some people carry rare mutations that make them predisposed to develop severe COVID-19, while more common polymorphisms can also substantially influence one’s risk as a result of infection.

Genomic tests could identify those who have genetically mediated resistance to COVID-19 and other infectious diseases. We use the term ‘genomic passports’ as a general term to refer to tools that can identify individuals who enjoy protection against a virus, due to the genomic variants they carry. During periods of ‘lockdown’, genomic passports could be used to allow immune individuals greater freedoms, perhaps permitting them to return to work, care for those at risk, visit friends and relatives or undertake other activities that expose them to the virus.^1^

There are currently many technical and practical obstacles to genomic passports, and they are unlikely to be a feasible tool used for the COVID-19 pandemic. Still, the possibility for their use for COVID-19 serves as a useful illustrative example of the ethical opportunities and risks of genomic passports more generally. The same ethical considerations that apply to using COVID-19 genomic passports will also apply to using genomic passports for future pandemics. Such pandemics will take place in the contexts of more advanced genomic technologies and more available sequence data. This will allow more rapid identification of genomic associations with infectious disease and the ability to identify those at low risk. Furthermore, growth in the direct-to-consumer genomic testing industry means private companies may soon offer testing for infectious disease related genomic variants directly to consumers. There is therefore a need to scrutinise the ethical issues raised by genomic passports and testing for infectious disease related variants more generally.

In this paper, we distinguish three hypothetical passports that could be issued as a result of genomic sequencing. We refer to these as *severity certificates*, *resistance certificates* and *infectivity certificates*. Using COVID-19 as an example, we discuss contexts where a passport mechanism to identify people based on genetic tests might be justified and highlight differences with immunity passports. We then discuss some ethical objections to such passport systems, including that they will exacerbate existing social inequalities.

### Severity certificate

Some individuals infected with SARS-CoV-2 are completely asymptomatic, while others develop severe respiratory failure. Even after taking demographic factors such as age and other risk factors such as pre-existing cardiovascular disease, obesity and diabetes mellitus into account, there remains significant variation in COVID-19 severity. Individual genetic differences have now been shown to contribute to this variation. Whole-genome sequencing of individuals who have been infected with the SARS-CoV-2 virus found those who develop life-threatening symptoms had an eightfold increase in the odds of having a loss of function variant in genes that influence the regulation of type 1 interferons compared with those who are asymptomatic or have mild symptoms. Type 1 interferons are an important component in the innate immune response.^14^ Another study identified a region on chromosome 3 that is associated with a 1.7-fold increase in the odds of requiring mechanical ventilation after infection with SARS-CoV-2, as well as earlier age at hospitalisation.^15^ This region has then been mapped to variants first introduced into the human gene pool via Neanderthal interbreeding 60 000 years ago.^16^ Further genome-wide association studies looking at COVID-19 outcomes have confirmed these results and uncovered further variants that impact COVID-19 severity.^17^

In addition to COVID-19, genetic host factors have been associated with differences in severity in many other infectious diseases including influenza, meningitis and pneumonia.^18^ Furthermore, some genetic variants are associated with better recovery after illness due to infectious disease.^19^

The idea of using genomic testing to identify those at low risk of severe disease as a result of COVID-19, and other infectious disease, is clearly plausible. Are there benefits in being able to identify such individuals?

It is important to note that those with a low genetic predisposition to develop severe COVID-19 may still carry the virus and transmit it to others. Indeed, so-called ‘asymptomatic super-spreaders’ are thought to have been a particularly important driver of the COVID-19 pandemic in its early stages.^20^ As infected individuals who show no signs of illness still pose a risk to others, restricting their movement is ethically justified.

There are scenarios, however, where it might be useful to identify individuals at low personal risk from infectious diseases. During periods of lockdown, essential workers continue to provide basic services such as healthcare, food and cleaning. Having mechanisms to distinguish essential workers who are at low risk as a result of infection may help better coordinate these essential activities and allow employees to make more informed decisions when at work. Individuals with ‘low severity’ passports could thus be given greater freedom to engage in high-risk activities, perhaps including assisting infected individuals. Indeed, it could be argued that those with genetic resistance have a positive moral obligation to provide assist others in this time.

Another circumstance where identifying those of low personal risk of disease will be useful is when distributing vaccines. As at the beginning of 2021, there are several approved vaccines for COVID-19. However, there are many practical challenges with ensuring distribution to all individuals in need of a vaccine, both within countries and globally. As a result, many nations are drawing up prioritisation guidelines, which dictate who will first get vaccinated as supplies become available. Many of these guidelines identify individuals who have increased ‘risk of serious, life-threatening complications from COVID-19’, as a priority group for early vaccination.^21^ This would include individuals who have underlying genetic susceptibility to COVID-19. A passport or certificate system based on individual genetic susceptibility to infectious disease could thus help guide vaccine distribution in cases where supply of a vaccine is limited.

In sum, there might be some circumstances where a passport or certificate system to identify individuals with low-severity genetic variants is useful. However, because these individuals still pose a risk to others, they are unlikely to be useful as means of identifying individuals who can be exempted from quarantine or lockdowns.

### Resistance certificates

Some people with exposures to the SARS-CoV-2 do not become infected.^22^ One possible reason is that specific genetic variants make it difficult for the virus to enter our cells. SARS-CoV-2 enters our cells through the ACE2 receptor, found in the lungs, arteries, heart, kidney and intestines. In vivo studies have demonstrated that variants in several genes that influence ACE2 can prevent SARS-CoV-2 from entering cells.^23^ It is possible that naturally occurring variants are present in the population and have the effect of making people naturally resistant to COVID-19.

It has long been established that some individuals carry genomic variants that make them immune to particular infectious diseases. One of the most well-known examples is the CCR5-Delta32 polymorphisms. CCR5 codes for a cell receptor were found in immune cells. Some people have a 32 base-pair deletion in this gene (called the delta32 variant), which alters the receptor in such a way that the HIV virus cannot enter the cells, and this protects against AIDS.^24^ Variants conferring cellular resistance to norovirus^25^ and malaria^26^ have also been identified.

This existence of people who are genetically resistant to pathogens has direct implications for the ethical justification for restrictive public health policies. The European Convention of Human Rights establishes a right to freedom of movement that cannot be restricted but ‘for the prevention of the spreading of infectious diseases’.^27^ It is very hard to justify keeping individuals under lockdown or in quarantine if they genuinely pose little or no risk to others. Indeed, it might be argued to be a breach of their fundamental rights.

A passport or certificate system that helps identify people who are genetically resistant infectious agents during a pandemic could therefore have many benefits. It could instantly benefit those with genetic immunity by permitting them greater freedom. This could help society more broadly as the genetically immune could provide assistance to others and function in other socially beneficial ways as previously outlined. More specifically, testing for genomic resistance among essential workers could improve the delivery of these services by shielding those must susceptible to the virus from exposure.

### Infectivity certificates

Just as there is individual variation in how resistant people are to infection by pathogens, there is variation in how likely individuals are to spread pathogens to others. Some recent studies indicate that ~70% of individuals infected with SARS-CoV-2 do not infect another person.^22^ It is plausible that some might carry genetic variants that make them less likely to infect others. To date, there has been little research looking at genetic associations between genetic variants and individual infectivity. However, there have been calls to genetically sequence so-called ‘super-spreaders’ of COVID-19 to look for genetic associations.^28^ Such research may also uncover variants that make people less likely to infect others.

If we had the ability to identify individuals with reduced infectivity, would we have reason to do it? Just as in the case of variants that made people resistant to infection, people of low infectivity would pose low risk to others. The benefit of placing these individuals under lockdown or in quarantine is far reduced. While they may still be at risk of personal harm from infection, preventing harm to one’s self falls short of meeting the legal justification for severely restricting an individual’s freedom of movement given in international law.^27^ As stated above, freedom of movement is widely considered an important human right, which may only be restricted to prevent widespread harm to others. Preventing someone’s movement from their own good, rather than to prevent harm to others, is a problematic instance of paternalism.

Furthermore, information regarding infectivity would be useful to know for essential workers. If some individuals pose lower risk to others, this is a reason to prioritise them for essential services.

It is interesting to think about vaccination policies in contexts where individuals have high infectivity and low personal susceptibility. In these cases, the private interests of individuals in getting vaccinated may be quite low, whereas the public interest in their vaccination is very high. These individuals may be unmotivated to get vaccinated as they personally stand to benefit little. Coercive vaccination policies have been defended as ethically justified where the following conditions are met^29^:

1. There is a grave threat to public health.
2. The vaccine is safe and effective.
3. Mandatory vaccination has a superior cost/benefit profile compared with other alternatives.
4. The penalties associated with non-compliance are proportionate.

On this view, if the above conditions are met, it would be ethically appropriate to use coercive policy measure to increase vaccination rates among individuals who have high infectivity but are at low personal risk of disease. This includes the withholding of government benefits and through offering financial incentives.

### Ethical issues

The idea of using genomic passports has much in common with the idea of immunity passports—a topic that has received heated debated in the bioethics literature.^27 30–33^ Many of the potential ethical concerns that have been highlighted for immunity passports could apply to genomic passports. Indeed, critics of immunity passports often object to any records that could distinguish immune from susceptible individuals. For example, when arguing against immunity passports, Kofler and Baylis^32^ state that ‘any documentation that limits individual freedoms on the basis of biology risks becoming a platform for restricting human rights, increasing discrimination and threatening — rather than protecting — public health’.

In our view, this statement gets things backwards. As we saw with COVID-19, in the absence of a vaccine, limiting movement is the only effective way to prevent the spread of a virus. These limitations can be justified only when an individual’s free movement poses a threat to others. As freedom of movement is a fundamental human right, immunity and genomic passports are mechanisms that protect, rather than restrict, human rights during public health crises.

Nonetheless, the use of genomic passports, like immunity passports, raises several ethical issues that need to be carefully considered. We summarise the different ethical issues raised by immunity and genomic passports in table 1.

**Table 1:** Comparison of ethical issues raised by genomic passports and immunity passports

Ethical issue	Immunity passport	Genomic passport
Incentivise infection	Yes (for natural immunity)	No
Privacy risk	Minimal personal information collected	Potentially substantial personal information collected
Undermine solidarity	Possible	Possible
Exacerbate inequalities	Possible	Likely if based on biased data
Fairness (luck egalitarianism)	No unique concerns	Raises unique concerns

One criticism of immunity passports is that they incentivise infection. During a pandemic, individuals who desire increased freedom will be motivated to become infected with a virus, in order to develop immunity and thus access an immunity passport.^30^ Such critiques do not apply to genomic passports. Nonetheless, genomic passport poses their own unique ethical issues, as well as raising some issue familiar to immunity passports. We will now outline some possible ethical issues raised by genomic passports, which fall under the headings of privacy, solidarity and inequality.

### Privacy

There is no doubt that genomic passports involve a loss of privacy in some sense. They are a tool to help distinguish between individuals on the basis of personal information (whether or not they have specific genetic variants.) However, we often accept losses of privacy if it helps facilitate something beneficial, such as our free movement (as exemplified by travel passports).

Some worry that immunity passports will be a gateway to more extensive tracking of personal information and control of public spaces.^32^ These worries will be exemplified in the case of genomic passports. In addition to revealing information about someone’s susceptibility to infectious disease, genomic sequencing can potentially reveal lots of other types of information about someone, including their ethnicity and predisposition to health and character traits.

A common feature of science fiction novels is totalitarian governments who use technologies to overtly control the lives of everyday citizens.^2^ Genomic passports may be portrayed as a step towards such dystopias, where all privacy protections have been lost. Once a system is in place that controls individual movement based on genomic characteristics, it may lead to the creation of central databases of genomic information and forms of tracking that persist after a pandemic has passed.

However, this is a very long bow to draw. It is clearly conceivable that liberal democracies can use genomic passports to promote the collective good in ways that are consistent with protecting individual privacy. There are several measures that could be implemented to reduce the privacy-related risks associated with genomic passports and indeed immunity passports. For one, we could ensure that any passport scheme was purely voluntary and that individuals were not compelled to divulge private information. Second, we could ensure that private information that was required to issue the passport was not stored on a publicly available database and not used for further surveillance. Third, we could ensure that analysis is done for the sole purpose of generating information about pathogen resistance and that any subsequent analysis was entirely at the behest and control of individuals. Fourth, we could adopt clear provisions for data storage, protection, sharing and destruction. Therefore, while the privacy implications of genomic passports are significant, they do not decisively count against their use.

### Solidarity

Part of the effectiveness of public health measures, such as social distancing, wearing masks and lockdowns, depend on a sense of solidarity. Individual sacrifices are worth it, because others are also making sacrifices, and collectively, this promotes the greater good. If some people who are at low personal risk of disease stop making sacrifices, this may undermine the collective camaraderie essential for successful collective health measures. If we allow those who are genetically resistant greater freedoms, this might undermine the message that we are ‘all in this together’. Behavioural scientists have suggested that these concerns carry weight, as they are consistent with our tendency to view things in terms of an ‘in-group’ and ‘out-group’ mentality.^34^

However, such concerns are speculative. We can think of ways in which allowing greater freedoms to those who are genetically resistant might make it easier for others to commit to extended lockdowns. If a small portion of the population is permitted greater freedoms, they could help others with essential tasks and lessen the burden of being confined to their homes. In this way, genomic passports might enable more acts of prosocial altruism.^35^ During the COVID-19 pandemic, we have seen many instances of individuals trying to help those who are less fortunate, from individuals donating plasma, helping with research studies and taking on riskier roles at work. By providing a mechanism for individuals to help each other, genomic passports may help communities function together, rather than undermining solidarity.

### Inequality

Another critique of immunity passports is premised on the idea that will exacerbate existing socioeconomic inequalities.^32^ The relatively well-off have greater access to health resources and are more likely to be able to access immunity tests than those in lower socioeconomic classes. A passport system might thus only provide benefits to the relatively well-off and do nothing to improve the standing of the worst off. Such considerations indicate it would be unethical to employ an immunity passport system, unless measures to ensure fair access were also implemented.

However, genomic passports would raise for more fundamental concerns. Those who live in high-income countries, and moreover those of higher socioeconomic status in high-income countries, are much more likely to participate in genomic research.^36^ As a result, genomic databases tend to be heavily skewed with data from those of European descent. For example, one of the most widely used resources for genetics research, the UK Biobank, contains samples from over half a million people, 94% of whom report having a ‘white’ ethnic background.^37^ This has sometimes led to the failure of identified risk variants to translate to those of other races, with sometimes devastating consequences.^38^

Likewise, if we identify resistance variants primarily in those of European descent, this stands to primarily benefit those of European ancestry. Those with non-European ancestry are typically the worst affected by COVID-19.^39^ A passport system that was implemented based on studies in Europeans and without consideration of broader social justice could therefore exacerbate the differences between groups based on ethnicity.^40^

This shows the importance of current initiatives that aim to increasing diversity in genomic research.^41^ It is essential that any genomic associations that underlie host–genome interactions be investigated in diverse populations before they are used as a basis for policy. More broadly, it is imperative that the consequences of any genomic passport system on existing inequalities be thoroughly investigated before they are implemented.

### Fairness

Another objection that applies more significantly to genomic, rather than immunity, passports draws on the philosophical view of ‘luck egalitarianism’. Luck egalitarianism is a family of views in distributive justice that aim to counteract the effects of brute luck on people’s lives. According to this view, justice demands that differences in how well-off people should be wholly determined by the responsible choices people make, rather than brute luck. People should not enjoy advantages just because they happen to be born that way.

Genomic passports offer privileges to people purely on the basis of their genes. Many would see it as a very dangerous precedent to directly afford greater social advantages to people because of their biological make-up. Those born with variants that make them less susceptible to infectious disease are already lucky. Giving them greater freedoms just increases their advantages.

Acquired immunity and vaccination seem different in this sense. In the case of acquired immunity, someone might deserve greater freedom because they have already suffered, and those who are vaccinated might deserve greater freedoms because they have taken on the risks of vaccination and contributed to herd immunity through their choices. Genomic passports therefore raises specific concern regarding luck egalitarianism.

While it is plausible that we should not award people with extra advantages on the basis of inherited characteristics, another plausible principle is that we should not harm people, purely for the sake of making everyone more equal. Levelling down equality occurs when we impose a harm on well-off individuals, for the sole purpose of making them worse-off, in order to improve equality. It is widely considered to be impermissible in moral philosophy.^42^

Confining people in a room or house, even though they pose no harms to others, can be seen as a form of levelling down equality. It is in effect making those with genetic resistance against a pathogen worse off, merely for the sake of fairness or equality.

Importantly, the use of genomic passports by the resistant may also benefit the more susceptible. Passports provide a way for those at low personal risk to assist with essential tasks and lessen the burden for others confined in their homes. The claim that genomic passports would be unfair is thus inconsistent with a Rawlsian conception of fairness as articulated through the ‘veil of ignorance’ thought experiment.^43^ If no one in society knew ahead of time who would be resistant to a virus and who would not be, it seems likely that many would agree to a system where the resistant was granted greater freedoms under conditions of lockdown. Another way of expressing this point is through reference to Rawls’s ‘difference principle’, which argues that inequalities are only acceptable if they help raise the standard of the worst-off.^43^ Allowing the genetically resistant more freedoms can meet this standard, as having some individuals free to assist others benefits everyone.

## Conclusion

The COVID-19 pandemic has highlighted the now rapid speed in which genomic research can take place. Just 3 months after the WHO first classified COVID-19 as a pandemic, the first genome-wide association study for COVID-19 was published. Future pandemics will take place in the context of even more advanced genomic technologies.

One-way genetic information can be useful during a pandemic is helping identify individuals who pose little or no risk to others. In the absence of a vaccine, restrictive public health measures such as lockdowns are the most effective way to slow the spread of a virus. However, these measures disproportionately harm those of lower socioeconomic status who are already the worst off. The use of a genomic passport system to identify those who pose no risk to others could help rectify this injustice. Such systems can also benefit society more broadly, as it enables those who are genetically resistant to help others and perform essential services.

The use of genomic passports raises both familiar and distinct ethical issues. The most serious concern is inequality. Genomic research has a history of being skewed towards those of European descent and higher socioeconomic status. If we do not look for genomic association in diverse populations, it is possible that a passport system would only benefit those who already comparatively well-off and further entrench existing social inequalities. This emphasises the need for any passport system to be based on unbiased, representative genomic data.

## Footnotes

Rather than giving the genetically resistant extra freedoms, another possibility raised by identifying genetic variations in response to infectious disease is putting greater restrictions on those who are more susceptible to severe disease. While this also raises interesting ethical questions, we do not consider them here for reasons of manuscript length. We would like to thank an anonymous reviewer for altering us to this possibility.

For example, the Oceanian regime in George Orwell’s *1984*, and the Panem state in the series *The Hunger Games.*
